# Exosomal Non-Coding RNAs: New Insights into the Biology of Hepatocellular Carcinoma

**DOI:** 10.3390/curroncol29080427

**Published:** 2022-07-29

**Authors:** Qian Zhang, Hanlin Li, Yang Liu, Jian Li, Chunling Wu, Hua Tang

**Affiliations:** 1 School of Basic Medical Sciences, Southwest Medical University, Luzhou 646000, China; 20200199120025@swmu.edu.cn (Q.Z.); 20210199120032@stu.swmu.edu.cn (H.L.); 20210199120033@stu.swmu.edu.cn (Y.L.); 2 School of Basic Medical Sciences, Chengdu University, Chengdu 610106, China; lijian01@cdu.edu.cn; 3 Central Nervous System Drug Key Laboratory of Sichuan Province, Luzhou 646000, China; 4 Engineering, Informatics Fusion and Transformation Key Laboratory, Luzhou 646000, China

**Keywords:** exosome, hepatocellular carcinoma, long non-coding RNA, microRNA, tumor microenvironment

## Abstract

Exosomes, extracellular vesicles with a diameter of 40 to 160 nm, are among the smallest extracellular vesicles released by cells. They deliver different cargoes, including proteins, DNAs, and RNAs, and facilitate communication between cells to coordinate a variety of physiological and pathological functions. Hepatocellular carcinoma (HCC) is the sixth common malignant tumor and the fourth leading cause of cancer-related death worldwide. Its molecular mechanism remains largely unknown, and there is a lack of reliable and noninvasive biomarkers for early diagnosis and prognosis prediction. Mounting evidence has shown that exosomes carry a variety of ncRNAs, such as long non-coding RNAs (lncRNAs), microRNAs (miRNAs), and circular RNAs (circRNAs), which play critical roles in the occurrence and progression of HCC. In this review, we summarize the recent findings of exosomal miRNAs, lncRNAs, and circRNAs in HCC from their impact on the development of HCC to their potential applications in the diagnosis and treatment of HCC.

## 1. Introduction

Cells of both prokaryotes and eukaryotes can form and release cellular vesicles to carry out their physiological functions. In 2014, the International Society for Extracellular Vesicles (ISEV) adopted “extracellular vesicles” (EVs) as a generic term for naturally released particles from cells, including exosomes, microvesicles, apoptotic bodies and other EV subsets [1]. Among them, exosomes are vesicles with a diameter of 40 to 160 nm (average ~100 nm) that originate from endosomes [2]. Owing to the similar size between exosomes and other EVs, theoretically and technically separating them is quite challenging. Yet, exosomes display distinct roles both in physical and pathological process.

Depending on the cells from which the exosomes are derived and the physiological or pathological processes involved, exosomal contents vary widely from DNAs or RNAs to proteins or lipids [3]. Upon formation, endosomes are secreted out of cells by exocytosis to play central roles in cell–cell communication [4]. With the increase in the number of studies on exosomes, it has been reported that cancer-derived exosomes are closely related to the occurrence and development of cancer. Exosomal communication between different types of cells can support the proliferation of tumor cells [4], and the pivotal role of tumor-derived exosomes in the tumor microenvironment (TME) has been confirmed [5]. A myriad of experimental studies has indicated the great potential for the application of exosomes in the diagnosis, treatment, and prognosis of tumors [6]. 

Hepatocellular carcinoma (HCC) is a common type of tumor that threatens human health. According to the latest statistics in 2022, among the leading types of cancers, deaths related to liver and intrahepatic bile duct cancers rank fifth in males and seventh in females [7]. Numerous studies have shown that chronic hepatitis B (CHB) and C (CHC), alcohol addiction, as well as metabolic liver disease (especially non-alcoholic fatty liver disease (NAFLD)) are risk factors for HCC [8]. Early diagnosis and HCC monitoring can increase the possibility of a cure and improve the overall survival (OS) rate, albeit to a certain degree [9]. For instance, liquid biopsy can be used as an important means of the early diagnosis of HCC [10]. According to previous studies, the most widely used biomarker is alpha-fetoprotein (AFP, threshold of 20 ng/mL), which shows a low sensitivity of 40–60% and a high specificity of 80–90% [9]. Despite the high specificity of AFP, the low sensitivity of AFP puts forward a higher demand for the early diagnosis of HCC. Thus, cancer-derived exosomes are a worthy research direction [11,12], as they may lead to the development of novel diagnostic and therapeutic approaches for improving the clinical outcomes of HCC.

Currently, HCC-related exosomal ncRNAs have been reviewed in a few papers, yet we provided a updated insight into this issue by deep mining the latest literatures and quantitatively assessing the exomal diagnostic biomarkers. Furthermore, based on the functions and roles of exosomal ncRNAs in HCC, we summarize the current knowledge on the contribution of exosomal-related ncRNAs in HCC to TME (e.g., crosstalk between tumor and non-tumor cells, tumor angiogenesis, and tumor metastasis) and tumor resistance.

## 2. Exosome Biology

Exosomes were first noticed by Peter Wolf when he discovered that red blood cells can release vesicles and that these extracellular vesicles have anticoagulant activity [13]. In subsequent studies, Rose Johnstone officially named these vesicles “exosomes” [14]. Exosome biology plays a critical role in a variety of human diseases. Presently, there are some notable research findings on the mechanism of exosome formation, but there are still many unknown processes that remain unexplored.

**Figure 1:**
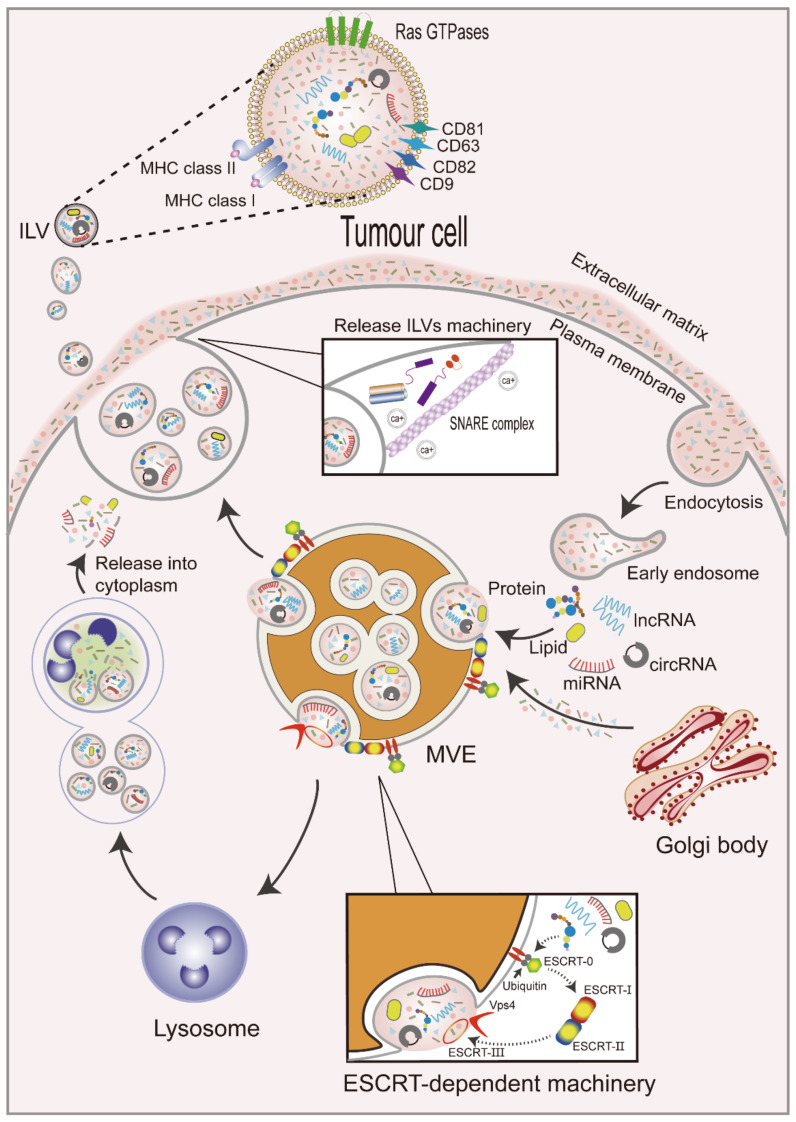
Biogenesis of exosomes. Extracellular components are internalized along with the plasma membrane to form early endosomes. Exosomal cargoes enter early endosomes to form multivesicular bodies (MVBs) through an ESCRT-dependent mechanism, which is shown in this figure. MVBs bind with lysosomes and release cargoes from vesicles into the cytoplasm, or under the actions of Rab GTPases, SNARE, and calcium (Ca^2+^), they fuse with the plasma membrane and release ILVs into the extracellular matrix. Exosomal surface proteins include Rab GTPases, tetraspanins, and MHC class I and II molecules.

The formation of exosomes occurs as follows (Figure 1). First, the plasma membrane internalizes extracellular components by endocytosis to form early endosomes [15]. Exosomal membrane cargoes are internalized by the plasma membrane or the Golgi apparatus into the endosome and are subsequently classified as intraluminal vesicles (ILVs) during endosomal maturation [16]. Early endosomal membranes are further enfolded to form multivesicular endosomes (MVEs) that contain ILVs [17], whose cargoes selectively or passively integrate a variety of intracellular materials, such as RNAs (including mRNAs, miRNAs, and other ncRNAs), DNAs, and lipids, into vesicles [18]. Cargoes aggregate and MVE transport in ILVs can occur by endosomal sorting complexes required for transport (ESCRT) dependent machinery or the ESCRT-independent machinery [19]. The ESCRT-dependent mechanism involves four complexes. ESCRT-0 is comprised of two subunits and recruits exosomal cargoes through ubiquitination. ESCRT-I is recruited by ESCRT-0 and then transported to the membrane. ESCRT-I interacts with ESCRT-II to form buds [20,21]. Lastly, ESCRT-III cleaves buds to form ILVs and promote the shedding of MVEs. The polymerized ESCRT-III complex can be dissociated from the MVE membrane by the energy provided by the sorting protein Vps4 [22]. However, several studies have revealed some ESCRT-independent mechanisms for exosomal cargo sorting, indicating that the mechanisms are more extensive and complex. 

Subsequently, MVEs can be fused with lysosomes for degradation [23] or plasma membranes for the release of ILVs, which are called exosomes, into the extracellular space to carry out their physiological functions [24]. This process is regulated by Rabs and other Ras GTP enzymes, soluble N-ethylmaleimide-sensitive factor attachment protein receptor (SNARE) proteins and their regulators [25], and intracellular Ca^2+^ [26]. Rab GTPases are the largest class of small GTPases involved in the regulation of MVE maturation and targeting to the plasma membrane [27]. For example, Rab27a and Rab27b have been demonstrated to participate in the docking of MVEs to plasma membranes [28], and Rab27a can be inhibited by KIBRA to control exosome release [29]. Rab GTPases are located in exosomal membranes, and tetraspanins (CD9, CD63, CD81, and CD82) [30,31] and major histocompatibility complex (MHC) class I and II molecules [32] have also been reported to be enriched, playing important roles in the membranes of exosomes. SNARE proteins promote the membrane fusion of vesicles with plasma membranes or different organelles [33]. Furthermore, increased intracellular Ca^2+^ levels in human erythrocytic leukemia K562 cells treated with monensin (Na+/H+ exchanger that induces intracellular changes) resulted in increased exosome release [34].

## 3. Non-Coding RNAs 

RNAs are divided into coding RNAs (mRNAs) and non-coding RNAs based on whether they can encode proteins. Studies have shown that mRNAs account for less than 2% of the total genome, and more than 98% are ncRNAs, such as lncRNAs, miRNAs, circRNAs, small nucleolar RNAs (snoRNAs) [35], tRNA-derived small RNAs [36], and piwi-interacting RNAs [37,38]. At a time when ncRNAs were poorly understood, they were considered nonfunctional “junk” [39]. However, with further research, ncRNAs have been found to not only regulate basic biological processes, such as growth, development, and organ function, but also appear to play key roles in a wide spectrum of human diseases, especially cancer [40]. Extensive alterations in ncRNA processing factors or ncRNAs themselves can affect multiple aspects of tumorigenesis [41]. Exosomes are intracellular vesicles secreted by most cells, and ncRNAs have been detected in exosomes isolated from the body fluids of cancer patients. For instance, Yu et al. extracted exosomes from serum samples of 79 HCC patients and confirmed that circulating exosomal ncRNAs (miRNA-21 and lncRNA-ATB) were associated with the TNM stage and other prognostic factors. These results suggest that exosomal miRNA-21 and lncRNA-ATB are new prognostic markers and therapeutic targets for HCC [42]. 

## 4. Functions of Exosomal miRNAs in HCC

MiRNAs are ncRNAs with a length of 21–22 nucleic acids that regulate gene expression by recognizing homologous sequences and interfering with transcription, translation, or epigenetic processes [43]. Multiple genes in the same cellular signaling pathway can be simultaneously targeted by a certain miRNA [44]. Complex interactions between miRNAs and other RNAs, including mRNA, lncRNA, circRNA and pseudogenes, have been found [45]. RNAs or pseudogenes that communicate with each other can regulate other RNAs’ transcripts through competing the shared miRNAs, known as competing endogenous RNAs (ceRNAs) [46]. MiRNA regulates mRNA expression by matching with the 3′-UTR of mRNA [47], and exosomal miRNA also has the conserved function of targeting cancer-related genes’ 3′-UTR [48,49].

The increasing research on exosomal miRNAs secreted by HCC cells suggests that a further understanding of exosomal miRNAs in HCC is of great significance for diagnosis and treatment.

### 4.1. Exosomal miRNAs as Diagnostic Biomarkers for HCC

HCC shows poor prognosis and high risk of recurrence. Resection, liver transplantation, and chemotherapy are the primary treatment options for HCC, although there is still a lack of reliable biomarkers for the diagnosis of unresectable HCC [50]. Because exosomes can provide the specificity required of liver biopsy samples, as well as the non-invasive nature of peripheral blood samples, exosomal miRNAs have advantages in diagnosis, and they are potential biomarkers for liver disease. Further details are described in Table 1. Notably, the role of exosomal miRNAs as biomarkers cannot be substituted by serum-free miRNAs because exosomal miRNAs are more stable, owing to the encapsulation by lipid bilayers [15]. The expression of exosomal miRNAs is less vulnerable to the interferences from other blood components relative to that of serum-free miRNAs. Furthermore, miRNAs are selectively enriched in exosomes [51]. 

Despite the above advantages, there is still a long way to go before exosomal miRNAs can be used as biomarkers for clinical application. The isolation and purification of exosomes from blood will be a crucial step [52]. A variety of attempts have been made to separate exosomes with other EVs of a similar size, including supercentrifugation-based separation, density gradient centrifugation, polymer-based precipitation separation, microfiltration and sides-based exclusion separation, etc. The most commonly used method for separating exosomes is supercentrifugation [53]. After ultracentrifugation (100,000× *g*), relatively pure exosomes can be separated through additional sucrose gradient steps [54]. However, exosomes isolated by this method are still contaminated not only by non-vesicular macromolecules [55], but also the microvesicles and apoptotic bodies with a similar size [56]. 

**Table 1:** Exosomal miRNAs are biomarkers for HCC.

MiRNA	Source	Application	AUC	Sensitivity (%)	Specificity (%)	Reference
miR-10b-5P	serum	differential diagnosis of HCC and normal tissues	0.934	90.7	75	[57]
miR-466-5p, miR-4746-5p	serum	early diagnosis	0.947	81.8	91.7	[58]
miR-638	serum	predicts the survival rate of HCC patients	-	-	-	[59]
miR-320d	serum	differential diagnosis of HCC and normal tissues	0.8694	-	-	[60]
miR-146a	plasma	identifies patients with HCC and cirrhosis	0.80 ± 0.4	81 ± 13	58 ± 22	[61]
miR-122, miR-148a, AFP	serum	differential diagnosis of early HCC and cirrhosis	0.931	86.0	87.5	[62]
miR-125b	serum	predicts the recurrence rate of HCC patients	0.739	83.0	67.9	[63]
		predicts the survival rate of HCC patients	0.702	85.5	53.4	

Several studies have shown that certain differentially expressed exosomal miRNAs secreted by HCC cells can be used as potential biomarkers for the diagnosis of HCC. HCC is a highly malignant tumor, and early diagnosis can help patients with treatment and prolong survival. In a report by Hyo et al., exosomal miR-10b-5p (AUC: 0.934, sensitivity: 90.7%; specificity: 75.0%; cutoff value: 1.8-fold) was identified as a potential serum biomarker for the early diagnosis of HCC through the analysis of exosomes extracted from normal liver and HCC cells [57]. Another study identified the combined use of exosomal miR-466-5p and exosomal miR-4746-5p as more accurate serum biomarkers for the early diagnosis of HCC (AUC: 0.947, 95% confidence interval (CI): 0.889–0.980, sensitivity: 81.8%, and specificity: 91.7%) [58].

Some exosomal miRNAs can be used as novel serum biomarkers in patients with HCC. Shi et al. reported a decreased level of exosomal miR-638 in serum samples from patients with HCC, and a negative correlation of exosomal miR-638 with tumor size, vascular invasion, and TNM stage. In addition, overexpression of miR-638 significantly inhibited the proliferation of Huh7 and SMCC7721 cells. HCC patients with higher serum exosomal miR-638 levels had longer OS, and low serum exosomal miR-638 levels were identified as an independent adverse prognostic factor for HCC [59]. Similarly, the significantly reduced level of exosomal miR-320d in the sera of HCC patients, as well as the culture media of HCC cell lines, could accurately distinguish HCC patients from healthy controls, and the low expression of exosomal miR-320d indicated the poor prognosis of HCC patients [60]. Furthermore, patients with cirrhosis were at high risk of developing HCC. Thorben et al. determined that exosomal miR-146a expression could separate HCC patients from cirrhosis patients, with an AUC of 0.80 ± 0.14 (sensitivity: 81 ± 13%; specificity: 58 ± 22%). Low expression of exosomal miR-16 was also associated with poor OS in patients with cirrhosis (*p* = 0.034) [61]. Wang et al. found that the combined use of serum exosomal miR-122, exosomal miR-148a, and AFP, with an AUC of 0.931 (95% CI: 0.857–0.973), could be used to distinguish early-stage HCC from cirrhosis [62].

Some miRNAs can be used as predictors of clinical disease recurrence and survival. Liu et al. found that miR-125b levels in exosomal samples were significantly higher in patients with CHB, cirrhosis, and HCC than in serum samples (*p* < 0.01, respectively). Moreover, compared with CHB patients (*p* < 0.01 and *p* = 0.06) and cirrhosis patients (*p* < 0.01), miR-125b levels in exosomal and serum samples of HCC patients were decreased. HCC patients with lower levels of exosomal miR-125b had shorter recurrence time (TTR) (*p* < 0.01) and OS (*p* < 0.01). Therefore, the levels of exosomal miR-125b can predict the postoperative recurrence and survival of HCC patients [63].

### 4.2. Effects of Exosomal miRNAs on Tumor Microenvironment, Angiogenesis, Invasion, Proliferation, and Metastasis

HCC cells and tumor growth-related cells release exosomes that not only enhance signaling between the TME and the tumor but also promote angiogenesis, invasion, proliferation, and metastasis (details in Table 2).

The tumor microenvironment consists of fibroblasts, endothelial cells, extracellular matrix (ECM), and other components. Several studies have indicated that abnormalities within the TME are the main cause of tumor progression and metastasis, and exosomal crosstalk between cells of the TME and tumor cells has been reported to be critical [64]. Cancer-associated fibroblasts (CAFs) are important components of the TME, and their interactions with cancer cells play a major role in mediating the formation and activation of CAFs. However, CAFs release a variety of regulatory factors to modulate the biological characteristics of tumor cells and other stromal cells, thereby affecting the occurrence and progression of tumors [65,66]. A recent study indicated that normal HSCs are transformed into CAFs after the action of HCC cell-derived exosomes. For example, HCC cell-derived exosomal miRNA-21 activates the PDK1/AKT signaling pathway in HSCs by downregulating the target PTEN and transforming HSCs into CAFs [67]. In turn, HSC-derived exosomal miRNA-335-5P can be delivered to HCC cells to inhibit tumor growth and invasion, thereby playing an antitumor role [68]. Another study showed that exosomal miR-320a derived from CAFs of HCC patients, combined with its downstream target PBX3, could inhibit the growth and migration of HCC cells [69]. Li et al. confirmed that mast cells (MCs) and tumor cells could interact through exosomal miRNAs. MCs were stimulated by the hepatitis C virus E2 envelope glycoprotein (HCV-E2), which increased exosomal miRNA-490 levels that affected HCC cells, thereby inhibiting the EGFR/AKT/ERK1/2 pathway, and ultimately suppressing tumor metastasis [70]. Wang et al. showed that the treatment or transfection of HCC cells with exosomal miR-125a and exosomal miR-125b from tumor-associated macrophages (TAMs) inhibited tumor cell proliferation and stem cell characteristics, which was associated with exosomal miR-125a/b targeting CD90 [71]. Another study reported that miR-146a-5p was regulated by the transcription factor SALL4 during the M2 polarization of macrophages [72]. Immune cells in the TME are also affected by HCC cell-derived exosomes. Researchers have demonstrated that HCC cell-derived exosomal miR-92b could suppress the expression of CD69 and the cytotoxicity of natural killer (NK) cells mediated by CD69, and ultimately lead to immune escape [73]. Similarly, Liu et al. showed that the effects of the endoplasmic reticulum (ER) stress on HCC cells promoted the release of exosomal miR-23a-3p. Exosomal miR-23a-3p could up-regulate the expression of PD-L1 in macrophages by inhibiting PTEN to activate the PI3K/AKT pathway, while the elevated level of PD-L1 in macrophages inhibited T-cell function and induced immunosuppression [74].

It is well known that solid tumors require an adequate blood supply to grow [75]. Tumor-induced angiogenesis, vascular invasion, and metastasis in cancer patients are contributing factors to the poor prognosis of patients with malignant tumors [76,77]. Many types of exosomes can affect angiogenesis. For example, Fang et al. reported that the stable expression of exosomal miR-103 in HCC cells weakened the integrity of endothelial junctions by targeting multiple endothelial junction proteins, thereby increasing vascular permeability and promoting tumor metastasis [78]. Recently, a novel biomarker miRNA was identified from a highly metastatic hepatic cell line (HuH-7M), where exosomal miR-638 secreted by HuH-7M cells could promote vascular permeability by inhibiting the endothelial expression of VE-cadherin and ZO-1 [79]. Wang et al. reported that elevated exosomal miR-1290 levels were found in the sera of patients with HCC, and miR-1290 promoted tumor angiogenesis by targeting SMEK1 [80]. In another study, miRNA-210 was delivered to endothelial cells, where it directly inhibited SMAD4 and STAT6 expression to enhance angiogenesis [81], while miRNA-378b promoted angiogenesis and HCC progression by targeting transforming growth factor β receptor III (TGFBR3) [82]. In addition to autonomously secreting miRNAs, HCC cells can also derive related exosomal miRNAs to affect angiogenesis, if they are affected by TAMs. Human liver stem-like cells (HLSCs) can derive a set of specific miRNAs, namely, miR-15a, miR-181b, miR-320c, and miR-874, which can inhibit the angiogenic properties of tumor-derived endothelial cells (TECs) [83]. Under hypoxia, exosomal miR-155 derived from HCC cells can affect angiogenesis [84]. Taken together, exosomal miRNAs can orchestrate various angiogenic and anti-angiogenic factors.

Cell proliferation, invasion, and metastasis are often the leading causes of cancer-related death. Many studies have reported that the proliferation, invasion, and metastasis of HCC cells are closely related to the aberrant expression of miRNAs, such as miR-224, which can reduce the expression of glycine N-methyltransferase and promote tumor cell proliferation and invasion [85]. For example, miR-21 and miR-10b expression can be induced by the acidic environment of HCC to promote the proliferation, invasion, and metastasis of cancer cells [86]. The mechanism by which miR-21 promotes HCC cell growth is that it affects the expression of the tumor suppressor gene phosphatase and tensin homolog pseudogene 1 (PTENp1) and PTEN by regulating the expression of Tet methylcytosine dioxygenases (TETs) [87]. Yu and colleagues reported that patient-derived cell (PDC) cultures of HCC showed both rapid and slow cell migration, and differentially expressed miRNAs were identified in both rapid and slow cell migration groups. Interestingly, the target genes of these miRNAs were significantly enriched in the “focus adhesion” pathway. Further evidence shows that these miRNAs influence tumor metastasis [88].

The epithelial–mesenchymal transition (EMT) is a process of transformation from epithelial cells to mesenchymal cells. The EMT is closely related to tumor cell migration and invasion [89]. In one study, Lin et al. highlighted that differentially expressed miRNAs appeared in HCC cells after the EMT, compared with HCC cells in which low exosomal miR-374a-5p expression inhibited the proliferation and migration of HCC cells [90]. Another study demonstrated that an elevated exosomal miR-92a-3p level in highly metastatic HCC cells promoted the EMT in receptor cancer cells by targeting PTEN and activating AKT/Snail signaling [91]. In a study by You and colleagues, local hypoxia induced the production of exosomal miR-123f by HCC cells, and miR-123f, in turn, enhanced the proliferation, migration, invasion, and the EMT of HCC cells under low oxygen conditions by targeting LHX6 to inhibit the Wnt/β-catenin pathway [92].

The formation of pre-metastatic niches in the TME supports tumor cell growth. Studies have shown that normal fibroblasts are more easily transformed into CAFs by highly metastatic HCC cells. During this process, exosomal miR-1247-3p was secreted by highly metastatic HCC cells to directly target β-1,4-galactosyltransferases III (B4GALT3) and activate β1-integrin-NF-κB signaling in fibroblasts [93].

Autophagy plays a protective role in hepatocellular carcinogenesis. The mechanism of autophagy deficiency in HCC was studied through numerous mouse experiments. It was found that defects in autophagy affected the level of associated protein 9b (Atg9b) and caused ubiquitin-mediated protein accumulation, which rendered Atg9b-deficient hepatocytes susceptible to ER stress-induced cell death. Meanwhile, Atg9b-driven phagocytes may promote the docking of LC3 and p62 proteins to initiate autophagy-mediated degradation, while the absence of Atg9b prevents the recruitment of p62-related ubiquitin proteins for autophagosome–lysosomal degradation. Furthermore, tumor-derived exosomal miR-3091-3p was internalized by hepatocytes to inhibit the expression of Atg9b [94]. Thus, exosomal miRNAs are involved in biological events, leading to tumor proliferation, invasion, and metastasis. 

### 4.3. Effects of Exosomal miRNAs on Drug Resistance of HCC

Multiple miRNAs not only serve as biomarkers for the early diagnosis of HCC, but are also involved in drug resistance. Acquired drug resistance to targeted cancer therapy is challenging in that tumor cells initially respond to treatment but subsequently develop acquired drug resistance after long-term treatment [95,96]. Studying the mechanism of drug resistance during the treatment of HCC is helpful to the treatment and prognosis of patients. Currently, the drug therapies for HCC include 5-FU, sorafenib, and cisplatin (DDP). Fu et al. reported that multidrug-resistant cells (Bel/5-FU) activated the PI3K/AKT pathway to regulate angiogenesis and EMT by transferring exosomal miR-32-5p to HCC sensitive cells (Bel7402), thereby inducing the transformation of sensitive cells into drug-resistant cells [97]. In another study, the expression of exosomal miR-744 in the sera of HCC patients was lower than that in the sera of healthy individuals, demonstrating that low levels of miR-744 promoted the resistance of HCC cells to sorafenib and that PAX2 was a direct target of miR-744 [98]. In addition, resistance to DDP was not conducive to the prognosis of patients with HCC, and researchers have demonstrated that DDP resistance can be reversed by exosomal miR-199a-3p, thereby slowing the progression of HCC [99].

**Table 2:** Roles of exosomal miRNAs in HCC.

MiRNA	Expression	Source	Biological Function	Mechanism	Reference
miR-21	-	HCC cell	converts normal HSCs to CAFs	activates PDK1/AKT pathway in HSCs by targeting PTEN	[67]
miR-335-5p	-	HSC	inhibits tumor growth and invasion	downregulates the target of miRNA-335	[68]
miR-320a	-	CAF	inhibits growth and migration of HCC cells	targets PBX3	[69]
miR-490	-	MC	inhibits metastasis of HCC	inhibits EGFR/AKT/ERK1/2 pathway	[70]
miR-125a/b	-	TAM	inhibits HCC cell proliferation and stem cell characteristics	targets CD90	[71]
miR-146a-5p	-	M2 polarization of macrophage	promotes M2 polarization of macrophages	is regulated by SRLL4	[72]
miR-92b	↑	HCC cell	causes immune escape	inhibits CD69 and NK cytotoxicity	[73]
miR-23a-3p	↑	HCC cell	induces immunosuppression	inhibits PTEN activation of PI3K-AKT pathway	[74]
miR-103	-	HCC cell	increases permeability of proliferating vessels promotes metastasis	inhibits VE-cadherin expression in endothelial cells	[78]
miR-638	-	HCC cell	promotes vascular permeability	downregulates VE-cadherin and ZO-1	[79]
miR-1290	↑	serum	promotes angiogenesis	acts on the target SMEK1	[80]
miR-210	↑	HCC cell	promotes angiogenesis and progression of HCC	inhibits SNIAD4 and STAT6 expression in endothelial cells	[81]
miR-378b	↑	HCC cell	promote angiogenesis and progression of HCC	targets TGFBR3	[82]
miR-15a, miR-181b, miR-370c, miR-874	-	HLSC	inhibits growth of tumor cells	down-regulates FGF1 and PLAU	[83]
miR-155	↑ ^a^	HCC cell	promotes angiogenesis	-	[84]
miR-224	↑	HCC cell	promotes tumor cell invasion and proliferation	decreases glycine N-methyltransferase expression	[85]
miR-21, miR-10b	↑ ^b^	HCC cell	promotes proliferation, invasion, and metastasis of HCC cells	acts on the TETs/PTENp1/PTEN pathway	[86,87]
miR-374a-5p	↑ ^c^	HCC cell	promotes proliferation and migration of HCC cells	regulates GADD45A expression	[90]
miR-92a-3p	↑ ^d^	HCC cell	promotes EMT	targets PTEN; activates the AKT/Snail pathway	[91]
miR-1273f	↑^a^	HCC cell	enhances proliferation, migration, and invasion of HCC cells, as well as EMT	targets LHX6; inhibits the Wnt/β-catenin pathway	[92]
miR-1247-3p	↑ ^d^	HCC cell	promotes transfer niche formation	targets B4GALT3 activates β1-integrin-NF-κB pathway in fibroblasts	[93]
miR-3091-3p	-	HCC cell	promotes tumor autophagy deficiency	inhibits Atg9b	[94]
miR-32-5p	↑ ^e^	HCC multidrug resistant cell	induces multidrug resistance of HCC cells	activates the PI3K/AKT pathway	[97]
miR-744	↓	serum	increases sensitivity of HCC cells to sorafenib	targets PAX2	[98]
miR-199a-3p	-	HCC cell	reverses the resistance of HCC cells to DPP	-	[99]

HSC, hepatic stellate cell; CAF, cancer-associated fibroblast; MC, mast cell; TAM, tumor-associated macrophage; SALL4, Sal-like protein-4; VE-cadherin, vascular endothelial-cadherin; ZO-1, Zonula occludens-1; TGFBR3, transforming growth factor β receptor III; DPP, cisplatin; HLSC, human liver stem-like cell; ^a^ oxygen-deficient environment; ^b^ acidic environment; ^c^ epithelial–mesenchymal transformation of HCC cells; ^d^ highly metastatic HCC cells; ^e^ multidrug-resistant HCC cells; ↑, miRNA expression is increased; ↓, miRNA expression is decreased; -, miRNA expression is not described in the original study.

## 5. Functions of Exosomal LncRNAs in HCC

Currently, lncRNAs are a new class of ncRNAs comprised of more than 200 nucleic acids, which distinguishes them from other functional ncRNAs [100]. However, similar to other ncRNAs, lncRNAs regulate a variety of DNA sequence-specific transcriptional and epigenetic processes, including transcription initiation, splicing, processing, DNA and nucleosome modifications, isolation, and imprinting [101]. Studies indicate that they are regulatory RNAs that can be selectively packaged into exosomes to act as messengers for intercellular communication, thereby modulating tumor growth, metastasis, angiogenesis, and TME remodeling [102].

### 5.1. Exosomal LncRNAs as Diagnostic Biomarkers for HCC

Numerous studies have shown that exosomal lncRNAs have many advantages as diagnostic biomarkers for HCC (see Table 3 for details), which will be discussed in detail in the following sections.

Exosomal lncRNAs can be used as serum biomarkers for the early diagnosis of HCC. Researchers have confirmed that lncRNAs derived from small extracellular vesicles (EV-lncRNAs) in serum, such as EV-DLEU2, EV-HOTTIP, EV-MALAT1, and EV-SNHG1, can distinguish HCC patients from non-HCC patients. In addition, they have numerous advantages over AFP in the diagnosis of very early-stage HCC. In very early-stage HCC, the combined use of EV-MALAT1 and EV-SNHG1 provides the optimal biomarker panel [12]. Certain lncRNAs can also distinguish early-stage HCC patients from healthy individuals, as well as those with chronic hepatitis or cirrhosis. For example, Sun et al. evaluated exosomal biomarkers in the sera of 15 HCC patients and 15 healthy individuals by quantitative reverse transcription polymerase chain reaction (qRT-PCR). The results showed that the expression level of exosomal LINC00161 in the sera of HCC patients was significantly higher than that in healthy individuals (*p* = 0.011, fold change: 4.27) and was significantly correlated with serum AFP concentration and TNM stage. Therefore, exosomal LINC00161 may be a valuable biomarker for the diagnosis of HCC [103]. In addition, HCC patients and healthy individuals were distinguished by exosomal lncRNA SENP3-EIF4A1 expression (AUC: 0.8028) [104]. In a study by Xu and colleagues, the expression levels of two exosomal lncRNAs, ENSG00000258332.1 and LINC00635, were significantly higher in the sera of HCC patients than those in healthy individuals and patients with CHB (both *p* < 0.05). Receiver operating characteristic (ROC) curve analysis showed that ENSG00000258332.1 could distinguish HCC patients from CHB patients, and the area under the curve (AUC) was 0.719 (critical value: 1.345), while the AUC of LINC00635 was 0.750 (critical value: 1.690). When the two lncRNAs were combined with serum AFP (critical value: 20 μg/L), the AUC was 0.894, indicating that the combined use of ENSG00000258332.1, LINC00635, and AFP was optimal for the diagnostic and prognostic prediction of HCC patients [105]. Another study demonstrated that exosomal lncRNA-RP11-583F2.2 expression was higher in the sera of patients with HCC than in those with CHC and healthy individuals, and the optimal threshold for distinguishing serum parameters between HCC patients and non-HCC patients was ≥5.02, the sensitivity was 96.7%, and the specificity was 91.7% [106]. Similarly, exosomal lncRNA-HEIH was differentially expressed in CHC patients and hepatitis C virus (HCV)-associated HCC patients [107], and plasma exosomal lncRNA and RP11-85G21.1 (lnc85) were differentially expressed between HCC patients and healthy individuals [108]. Exosomal lncRNAs, as biomarkers, were also associated with the progression of HCC. For example, three differentially expressed lncRNAs, namely, ENSG00000248932.1, ENST00000440688.1, and ENST00000457302.2, were up-regulated in patients with metastatic HCC compared to those with non-metastatic HCC, suggesting that these lncRNAs can be used to monitor HCC metastasis [109]. In addition, Lee et al. reported that the high expression of exosomal lncRNA-ATB was an independent adverse factor for OS and progression-free survival [42].

### 5.2. Effects of Exosomal LncRNAs on the Tumor Microenvironment, Proliferation, Invasion, Metastasis, and Angiogenesis

Exosomal lncRNAs can not only serve as biomarkers for HCC, but can also remodel the TME, promote angiogenesis, and regulate the proliferation, invasion, and metastasis of HCC cells (see Table 4 for details). 

Intercellular crosstalk is one of the most important processes affecting tumor progression. It has been reported that cancer stem cell (CSC)-derived exosomes act on liver cancer cells to promote the expression of exosomal lncRNA TUC339, lncHEIH, and lncHOTAIR [110]. CD90^+^ HCC cells are characterized as invasive and metastatic CSCs. CD90^+^ HCC cells can release exosomal lncRNA H19, which is transported to human umbilical vein endothelial cells (HUVECs) to induce angiogenesis and influence intercellular adhesion, thereby affecting the TME. The mechanism of angiogenesis may be related to the increased mRNA expression of vascular endothelial growth factor (VEGF) and its receptor (VEGF-R1) by CD90^+^ HCC cell exosomes [111]. In a study by Li and colleagues, the depletion of YAP1 in endothelial cells resulted in an increase in exosomal MALAT1 released by these cells, which was transferred by exosomes into HCC cells to promote tumor cell invasion and metastasis by activating extracellular signal-regulated kinase 1/2 (ERK1/2) signaling [112]. Normal cells also secrete exosomes that affect HCC cells. Exosomal SENP3-EIF4A1-derived from normal cells can be transferred to HCC cells and inhibit tumor growth by competitively binding to miR-9-5p [104]. It has been reported that the ultra-conserved lncRNA TUC339 secreted by HCC cells can regulate cell proliferation and adhesion to the ECM [113]. Another study showed that exosomal TUC339 targets adjacent macrophages, modulating their activation and promoting their polarization to the macrophage (IL-4) phenotype, indicating that there is crosstalk between exosomal lncRNAs and tumor and immune cells [114]. Similarly, a study by Fan et al. revealed that exosomal PCED1B-AS1 sponges hsa-miR194-5p to increase the expression of PD-L1 and PD-L2 in recipient tumor cells, thereby inhibiting the expression of related genes in recipient T cells and macrophages and inducing immunosuppression [115]. Low oxygen environments also affect the progression of tumors. For example, researchers confirmed that exosomal linc-RoR is highly expressed, promoting tumor cell survival in hypoxic environments [116].

Presently, several researchers are focusing on the effects of tumor-derived exosomes on tumors. For example, Li et al. showed that exosomal lncRNA FAL1 expression was up-regulated in the sera of HCC patients and that the transfer of lncRNA FAL1 into HCC cells increased cell proliferation and migration [117]. Exosomal lncRNA-FAM138B derived from HCC cells inhibited the development of HCC cells by targeting miR-765 [118]. Secreted by tumor cells, exosomal lnc85 can bind and regulate miR-324-5p to promote the proliferation and migration of HCC cells [108], while exosomal LINC00161 can activate growth and metastasis-related signaling in HCC cells by targeting miR-590-3p and suppressing its downstream target ROCK2 [119]. The expression of lnc-FAM72D-3, as an oncogene, was up-regulated in HCC tissues, which enhanced cell survival and inhibited cell death. As a tumor suppressor gene, lnc-EPC1-4 was downregulated in HCC, which could enhance cell proliferation and inhibit cell apoptosis [11]. Ma and colleagues reported on the mechanism by which insufficient radiofrequency ablation (RFA) causes HCC tumor recurrence and metastasis. The study showed that the expression of exosomal lncRNA ASMTL-AS1 was highly expressed in HCC tissues, and the miR-342-3p/NLK/YAP signaling pathway could promote the malignancy of residual HCC after RFA deficiency [120]. Another study showed that HCC cells treated with propofol, a common intravenous anesthetic, reduced the expression of exosomal H19, which enhanced LIMK1 expression in HCC cells by sponging miR-520a-3p, thereby increasing the malignant potential of propofol-treated HCC cells [121].

### 5.3. Effects of Exosomal LncRNAs on Drug Resistance of HCC 

Several lncRNAs have been found to be involved in the drug resistance of HCC. Linc-VLDLR is an exosomal lncRNA that contributes to chemotherapeutic stress in HCC. HCC cells treated with multiple anti-tumor drugs can release more exosomal linc-VLDLR, and a study has shown that exosomal linc-VLDLR is a new medium for the development of resistance in HCC cells [122]. Researchers found that the highly expressed lncRNA ribonucleic acid-ROR (linc-ROR) in HCC cells was a stress-induced lncRNA that was enriched in exosomes. After HCC cells were incubated with sorafenib, the expression of linc-ROR was increased in HCC cells and released via exosomes, suggesting that EV-linc-ROR regulates the chemical sensitivity of HCC cells [123].

**Table 4:** Roles of exosomal lncRNAs in HCC.

LncRNA	Expression	Source	Biological Function	Mechanism	Reference
H19	-	CD90^+^ HCC cell	promotes angiogenesis	increases the expression of VEGF and VEGF-R1	[111]
MALAT1	-	-	promotes tumor cell invasion and metastasis	activates the ERK1/2 pathway	[112]
SENP3-EIF4A1	-	normal cell	inhibits tumor growth	competitively binds to miR-9-5p	[104]
TUC339	↑	HCC cell	promotes tumor cell proliferation and adhesion to extracellular matrix	siRNA inhibits TUC339	[113]
TUC339	↑	HCC cell	promotes macrophage polarization promotes the expression of M (IL-4) in macrophages	siRNA inhibits TUC339	[114]
PCED1B-AS1	↑	HCC cell	induces immunosuppression	sponges has-miR194-5P; enhances the expression of PD-L1 and PD-L2	[115]
linc-ROR	↑ ^a^	HCC cell	promotes the survival of HCC cells	P7OS6K1 phosphorylation (RPS6KB1), PDK1, and HIF-1α expression are decreased, and miR-145 expression is increased	[116]
FAL1	↑	serum	increases cell proliferation and migration	competitively binds to miR-1236	[117]
FAM138B	↓	HCC cell	inhibits proliferation, migration, and invasion of HCC cells	regulates miR-765	[118]
lnc85	↑	plasma	promotes proliferation and migration of HCC cells	binds and regulates miR-324-5p	[108]
LINC00161	↑	HCC cell	promotes the occurrence and metastasis of tumors	represses the activation of the ROCK2 pathway initiated by miR-590-3P	[119]
Inc-FAM72D-3	↑	HCC tissue	inhibits apoptosis	-	[11]
lnc-EPC1-4	↓	HCC tissue	inhibits apoptosis	-	
ASMTL-AS1	↑	HCC tissue	exacerbates the malignant behavior of HCC	acts on the miR-342-3P/NLK/YAP signaling pathway	[120]
H19	↓ ^b^	HCC cell	increases the malignant behavior of HCC cells	acts on the miR-520a-3P/LIMKT signaling pathway	[121]
linc-VLDLR	↑ ^c^	HCC cell	mediates drug resistance of HCC cells	-	[122]
linc-ROR	↑ ^d^	HCC cell	enhances the chemical sensitivity of HCC cells	-	[123]

^a^ Oxygen-deficient environment; ^b^ HCC cells treated with propofol (intravenous anesthetic); ^c^ HCC cells treated with a variety of antitumor drugs; ^d^ HCC cells treated with sorafenib; ↑, lncRNA expression is increased; ↓, lncRNA expression is decreased; -, lncRNA expression is not described in the original study.

## 6. Function of Exosomal CircRNAs in HCC

CircRNAs were first identified as viroids in RNA viruses in 1976 and are believed to be caused by endogenous RNA splicing errors [124]. They are a unique class of ncRNAs without 5′-3′ polarity and a circular structure with a polymeric acid tail [125]. It has been reported that circRNAs are enriched in exosomes, where they perform important biological functions by acting as competitive inhibitors of miRNAs to regulate protein translation and/or function [126]. It is worth noting that the high abundance, relative stability, and evolutionary conservation of circRNAs render them as likely candidates to exert biological effects in cancer progression [127,128]. Furthermore, exosomal circRNAs may participate in the pathophysiology of HCC.

### 6.1. Exosomal CircRNAs as Diagnostic Biomarkers for HCC

A study reported that exosomal circ_0070396 was differentially expressed in HCC patients compared with healthy individuals, suggesting that it can distinguish HCC patients from healthy individuals, as well as those with CHB and cirrhosis [129]. In another study, exosomal circRNAs served as biomarkers for predicting HCC recurrence and prognosis. qRT-PCR was used to measure the expression level of circAKT3 in exosomes in 124 HCC patients and 100 healthy subjects, and the results showed that the expression level of exosomal circAKT3 in HCC patients was significantly higher than that in healthy individuals. In addition, circAKT3 was found to be associated with a higher rate of tumor recurrence in HCC patients (hazard risk (HR): 3.14; 95% CI: 1.29–6.21; *p* = 0.012) and higher mortality (HR: 1.89; 95% CI: 1.04–3.01; *p* = 0.048) [130].

### 6.2. Effects of Exosomal CircRNAs on the Tumor Microenvironment, Proliferation, Invasion, Metastasis, and Angiogenesis

Several exosomal circRNAs have been reported to affect the TME, proliferation, invasion, metastasis, and angiogenesis (see Table 5 for more details).

A study by Huang et al. found that exosomal circRNA-100,338 was a mediator of signal transduction between HCC cells and endothelial cells. CircRNA-100,338 was overexpressed in the exosomes of highly metastatic HCC cells and could affect HUVEC proliferation, angiogenesis, and tight junctions between HUVECs to promote endothelial cell permeability [131]. Similarly, exosomal circCMTM3 expression was elevated in the sera of HCC patients and HCC cell lines, affecting the activity, migration, and invasion of HUVECs. Overexpression of circCMTM3 promotes angiogenesis by regulating SOX9 through sponging miR-3619-5p [132]. Exosomal circular de-ubiquitination (circ-DB) secreted by adipocytes also acts on HCC cells to promote tumor growth and reduce DNA damage. The mechanism was that the overexpression of exosomal circ-DB downregulates miR-34a, which in turn activates the expression of USP7 and cyclin A2, promotes tumor growth, and facilitates metastasis [133]. Circ-0051443 derived from normal cells was transported to HCC cells by exosomes, where BAK1 expression was upregulated by competitive binding with miR-331-3p, and this mechanism was utilized to promote apoptosis and halt the cell cycle [134]. Hsa_circ_0074854, which was upregulated in several HCC cell lines, was transferred to macrophages via exosomes, where it interacted with human antigen R (HuR) and promoted macrophage M2 polarization to enhance HCC invasion and migration [135]. In turn, normal liver cells were affected by exosomal circ_MMP2 of metastatic HCC cells, which formed a malignant phenotype and promoted HCC metastasis [136]. In addition, NK cells in the TME were also affected by exosomal circUHRF1. The secretion of circUHRF1 from HCC cells was transmitted to NK cells through exosomes, which reduced the secretion of IFN-γ and TNF-α by NK cells to promote immunosuppression [137].

Su et al. revealed that circRNA Cdr1as derived from HCC cells was mainly located in exosomes and directly bound to miR-1270, which upregulated AFP expression and stimulated the proliferation and migration of HCC cells. At the same time, circRNA Cdr1as stimulated the malignant behavior of surrounding normal cells through exosomes, contributing to the progression of HCC [138]. Similar studies have shown that in HCC, exosomal circPTGR1 secreted by cells with high metastatic potential could enhance the migration and invasion of these cells by interacting with miR449A-MET in weakly metastatic and non-metastatic cells [139]. Exosomal circTMEM45A was enriched in the sera of HCC patients, where it promoted tumor progression. CircTMEM45A acts as a sponge for miR-665 and modulates the direct interaction of miR-665 with insulin growth factor 2 (IGF2) [140]. In addition, circ_0061395 promotes the malignant behavior in HCC cells by regulating the miR-877-5p/PIK3R3 axis [141]. Lai et al. demonstrated that miR-338 inhibited HCC progression and glycolysis by targeting LRP6, and exosomal circFBLIM1 acted as a sponge for miR-338 to promote HCC progression [142]. Exosomal circ-ZNF652 was transferred to HCC cells, where it promoted cell proliferation, migration, and glycolysis. The binding of miR-29a-3p by circ-ZNF652 reduces the effect of GUCD1 on tumors [143].

### 6.3. Effects of Exosomal CircRNAs on Drug Resistance of HCC

Recent studies have shown that several circRNAs participate in the drug resistance of HCC patients. Xu et al. reported that the overexpression of circRNA-SORE was detected in sorafenib-resistant HCC cells and demonstrated the use of exosomes in the transmission of sorafenib resistance between HCC cells. Exosomal circRNA-SORE, upon transfer to HCC cells, can bind to YBX1 in the cytoplasm, block pre-mRNA processing factor 19 (PRP19)-mediated degradation to stabilize YBX1, and affect the expression of the downstream gene targets of YBX1 to transmit sorafenib resistance [144]. In addition, exosomal circ-G004213 promotes cisplatin sensitivity and improves cell survival. Circ-G004213 is a sponge of miR-513b-5p and regulates DDP sensitivity by modulating the interaction between miR-513b-5p and its downstream target gene PRPF39 [145].

Except for the exosomal miRNAs, lncRNAs and circRNAs, some other ncRNAs, including tRNA-derived small RNAs [36] and piwi-interacting RNAs [37,38], also participate in tumorigenesis. Due to the lack of adequate literature, we do not make too much of an elaboration here. 

**Figure 2:**
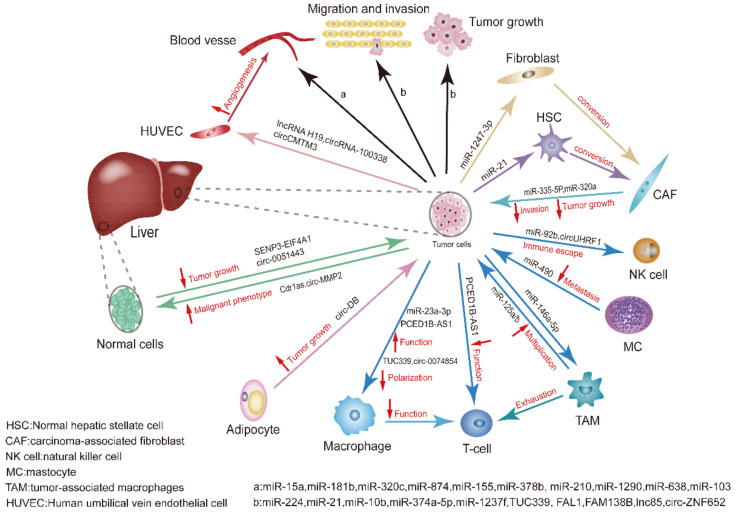
Summary of non-coding RNAs affecting HCC development. Exosomal ncRNAs mediate crosstalk between tumor cells and cells in the TME, where different types of cells interact with cancer cells, including infiltrating immune cells, CAFs, normal hepatic stellate cells (HSCs), adipocytes, and thin blood vessels that make up tumors. The complex multidirectional communication between these cells and tumor cells through exosomal ncRNAs promotes tumor growth, invasion, metastasis, and angiogenesis. The blue arrows indicate that the exosomal ncRNAs are originated from immune cells or acting on immune cells. The red up arrows show the function of promoting the corresponding exosomal ncRNAs, and red down arrows represent the function of inhibiting the corresponding exosomal ncRNAs. For example, adipocyte-stemmed circ-DB can facilitate tumor growth.

Generally, this review not only lists the roles of exosomal miRNAs, lncRNAs and circRNAs as biomarkers for HCC, but also illustrates the crosstalk between exosomal ncRNAs and TME as well as other cells in the process of tumor occurrence and development (Figure 2). 

Since there is no standardized evaluation system, we may obtain more reliable biomarkers to distinguish HCC from normal individuals by comparing the AUC of diagnostic models constructed from exosomal ncRNAs (Table 6). As shown, using combination of miR-466-5p and miR-4746-5p reached the highest AUC value of 0.947, which suggested the great possibility and reliability of these two exosomal miRNAs as biomarkers. However, it is crucial to note that such a comparison might be invalid when testing is not conducted on the same cohort. In addition, the standardized extraction of exosomal ncRNAs is the cornerstone of comparing the results of different studies. 

## 7. Conclusions

Exosomes are important tools for intercellular communication, and ncRNAs are transported between different tumor cells through exosomes. In recent years, scientific evidence has shown that miRNAs, lncRNAs, and circRNAs in HCC cell-derived exosomes have become novel biomarkers due to their high sensitivity, specificity and non-invasive detection, but there are still several factors to be considered. For example, how do researchers identify the best biomarkers, compare the results from different laboratories, and decide whether these biomarkers can be used to guide clinical treatment? Oncogenesis is a highly complicated process, and HCC progression depends not only on tumor cell-derived exosomes, but also on the influence of various cell-derived exosomes in the TME. Similarly, the drug resistance of tumor cells is also affected by exosomal ncRNAs, and studies on drug resistance mechanisms in HCC may improve the therapeutic benefits.

Although there is a myriad of research on exosomes, this field is still in its infancy, thus some further steps are needed to translate the existing findings into clinics: first, to break through the bottleneck of the extraction and purification method for exosome ncRNAs, namely, to ensure the corresponding biomarkers are from exosomes but not other EVs; second, to standardize the evaluation system of exosomal ncRNA biomarkers; third, more extensive clinical samples are required to validate the effectiveness of exosomal biomarkers; and the fourth, targeting the delivering of exosomes may be promising for drug delivery. In a word, the comprehensive understanding of exosomal ncRNAs will foster the early diagnosis, surveillance of recurrence, and prognosis prediction of HCC, as well as the improvement and optimization of clinical treatment. 

## Figures and Tables

**Table 3:** Exosomal lncRNAs serving as biomarkers for HCC.

LncRNA	Source	Application	AUC	Sensitivity (%)	Specificity (%)	Reference
MALAT1	serum	distinguishes between patients with and without HCC	0.908	92.063	81.579	[12]
SNHG1	serum	distinguishes between patients with and without HCC	0.898	80.769	85.246	
MALAT1, SNHG1	serum	early diagnosis of HCC	0.899	-	-	
LINC00161	serum	distinguishes between patients with and without HCC	0.794	-	-	[103]
SENP3-EIF4A1	plasma	distinguishes between patients with and without HCC	0.8028	-	-	[104]
ENSG00000258332.1	serum	distinguishes early HCC patients from CHB patients	0.719	71.6	83.4	[105]
LINC00635	serum	diagnosis of HCC	0.750	76.2	77.7	
ENSG00000258332.1, LINC00635, AFP	serum	diagnosis of HCC	0.894	83.6	87.7	
lncRNA-RP11-583F2.2	serum	distinguishes between patients with and without HCC	0.946	96.7	91.7	[106]
lncRNA-HEIH	serum-free and exosome	diagnosis of HCV-associated HCC	-	-	-	[107]
RP11-85G21.1	plasma	distinguishes between AFP^+^ HCC patients and non-HCC patients	0.883	80.5	76.5	[108]
	plasma	distinguishes between AFP^−^ HCC patients and non-HCC patients	0.869	80.0	76.5	
ENSG00000248932.1, ENST00000440688.1, ENST00000457302.2	plasma	monitors HCC metastasis	0.870	-	-	[109]
lncRNA-ATB	serum	predicts OS in HCC patients	-	-	-	[42]

**Table 5:** Roles of exosomal circRNAs in HCC.

CircRNA	Expression	Source	Biological Function	Mechanism	Reference
circRNA-100,338	↑ ^a^	plasma	promotes vascular permeability	-	[131]
circCMTM3	↑	blood	promotes angiogenesis	regulates the miR-3619-5p/SOX9 axis	[132]
circ-DB	-	adipocytes	promotes tumor growth and metastasis	inhibits miR-34a activates A2USP7/cyclin A2	[133]
circ-0051443	-	normal cell	promotes apoptosis and halts cell cycle progression	competitively binds to miR-331-3p upregulates the expression of BAK1	[134]
hsa_circ_0074854	↑	HCC cell	promotes the migration and invasion of HCC cells	interacts with HuR and promotes M2 polarization of macrophages	[135]
circ_MMP2	-	HCC cell	promotes the malignant behavior of normal hepatocytes	regulates the miR-136-5p/MMP2 axis	[136]
circUHRF1	↑	HCC cell	promotes immunosuppression	acts on NK cells, reduces the secretion of IFN-γ and TNF-α	[137]
Cdr1as	↑	HCC cell	promotes the proliferation and migration of HCC cells	spongy miR-1270 promotes AFP expression	[138]
circPTGR1	↑	high metastatic potential HCC cell	enhances the migration and invasion of cells with low or no metastatic potential	interacts with miR449a-MET	[139]
circTMEM45A	↑	serum	promotes the progression of HCC	regulates the miR-665/IGF2 axis	[140]
circ_0061395	↑	HCC tissue, serum, cell, and serum-derived exosome	promotes the progression of HCC	regulates the miR-887-5p/PIK3R3 axis	[141]
circFBLIM1	↑	serum	promotes the progression of HCC; promotes the glycolysis of HCC cells	regulates the miR-338/LRP6 axis	[142]
circ-ZNF652	↑	serum and HCC cell	promotes cell proliferation, migration, invasion; promotes the glycolysis of HCC cells	regulates the miR-29a-3p/GUCD1 axis	[143]
circRNA-SORE	↑ ^b^	HCC cell	promotes sorafenib resistance	blocks the degradation of YBX1 by PRP19	[144]
circ-G004213	↑ ^c^	plasma	increases the sensitivity of HCC cells to CIS	regulates the miR-513b-5p/PRPF39 axis	[145]

HuR, human antigen R; CIS, cisplatinum; ^a^ highly metastatic HCC cells; ^b^ Sorafenib-resistant HCC cells; ^c^ HCC cells treated with transarterial chemoembolization (TACE); ↑, circRNA expression is increased; -, circRNA expression is not described in the original study.

**Table 6:** The AUC of exosomal ncRNAs for the diagnosis of HCC.

ncRNAs	Source	Application	AUC	Sensitivity (%)	Specificity (%)	Reference
miR-466-5p, miR-4746-5p	serum	early diagnosis	0.947	81.8	91.7	[58]
lncRNA-RP11-583F2.2	serum	distinguishes between patients with and without HCC	0.946	96.7	91.7	[106]
miR-10b-5P	serum	differential diagnosis of HCC and normal tissues	0.934	90.7	75	[57]
miR-122, miR-148a, AFP	serum	differential diagnosis of early HCC and cirrhosis	0.931	86.0	87.5	[62]
MALAT1	serum	distinguishes between patients with and without HCC	0.908	92.063	81.579	[12]
MALAT1, SNHG1	serum	early diagnosis of HCC	0.899	-	-	[12]
SNHG1	serum	distinguishes between patients with and without HCC	0.898	80.769	85.246	[12]
ENSG00000258332.1, LINC00635, AFP	serum	diagnosis of HCC	0.894	83.6	87.7	[105]
RP11-85G21.1	plasma	distinguishes between AFP^+^ HCC patients and non-HCC patients	0.883	80.5	76.5	[108]
miR-320d	serum	differential diagnosis of HCC and normal tissues	0.8694	-	-	[60]
RP11-85G21.1	plasma	distinguishes between AFP^−^ HCC patients and non-HCC patients	0.869	80.0	76.5	[108]
miR-146a	plasma	identifies patients with HCC and cirrhosis	0.80 ± 0.4	81 ± 13	58 ± 22	[61]
SENP3-EIF4A1	plasma	distinguishes between patients with and without HCC	0.8028	-	-	[104]
LINC00161	serum	distinguishes between patients with and without HCC	0.794	-	-	[103]
LINC00635	serum	diagnosis of HCC	0.750	76.2	77.7	[105]

## Data Availability

Not applicable.
